# Characteristic expression of HTLV-1 basic zipper factor (HBZ) transcripts in HTLV-1 provirus-positive cells

**DOI:** 10.1186/1742-4690-5-34

**Published:** 2008-04-22

**Authors:** Tetsuya Usui, Katsunori Yanagihara, Kunihiro Tsukasaki, Ken Murata, Hiroo Hasegawa, Yasuaki Yamada, Shimeru Kamihira

**Affiliations:** 1 Department of Laboratory Medicine Nagasaki University Graduate School of Biomedical Sciences, Nagasaki City, Japan; 2 Department of Hematology, Nagasaki University Graduate School of Biomedical Sciences, Nagasaki City, Japan

## Abstract

**Background:**

HTLV-1 causes adult T-cell leukemia (ATL). Although there have been many studies on the oncogenesis of the viral protein Tax, the precise oncogenic mechanism remains to be elucidated. Recently, a new viral factor, HTLV-1 basic Zip factor (HBZ), encoded from the minus strand mRNA was discovered and the current models of Tax-centered ATL cell pathogenesis are in conflict with this discovery. HBZs consisting of non-spliced and spliced isoforms (HBZ-SI) are thought to be implicated in viral replication and T-cell proliferation but there is little evidence on the HBZ expression profile on a large scale.

**Results:**

To investigate the role of HBZ-SI in HTLV-1 provirus-positive cells, the HBZ-SI and Tax mRNA loads in samples with a mixture of infected and non-infected cells were measured and then adjusted by dividing by the HTLV-I proviral load. We show here that the HBZ-SI mRNA level is 4-fold higher than non-spliced HBZ and is expressed by almost all cells harboring HTLV-1 provirus with variable intensity. The proviral-adjusted HBZ-SI and Tax quantification revealed a characteristic imbalanced expression feature of high HBZ and low Tax expression levels in primary ATL cells or high HBZ and very high Tax levels in HTLV-1-related cell lines (cell lines) compared with a standard expression profile of low HBZ and low Tax in infected cells. Interestingly, according to the mutual Tax and HBZ expression status, HTLV-1-related cell lines were subcategorized into two groups, an ATL cell type with high HBZ and low Tax levels and another type with high Tax and either high or low HBZ, which was closely related to its cell origin.

**Conclusion:**

This is the first comprehensive study to evaluate the mutual expression profile of HBZ and Tax in provirus-positive cells, revealing that there are quantitative and relative characteristic features among infected cells, primary ATL cells, and cell lines.

## Introduction

Adult T-cell leukemia (ATL) is a unique T-cell malignancy derived from T-cells infected with a retrovirus of human T-cell leukemia virus type-1 (HTLV-1) [1-3]. ATL is clinically and hematologically characterized to develop step by step through smoldering, chronic, and acute stages after a long latency of HTLV-1 infection, revealing that ATL is a good experimental model of multi-step carcinogenesis.

Although it is a fact that HTLV-1 reaches an oncogenic event and causes ATL, the oncogenic mechanism of HTLV-1 is not fully understood. The HTLV-1 genome, in addition to the structural and enzymatic proteins gag, pol, and env, encodes the regulatory and accessory proteins tax, rex, p12^I^, p13^II^, and p30^II ^[4,5]. Among these viral proteins, Tax, encoded by pX in a double splicing manner, is thought to be mainly implicated in the oncogenesis of ATL via indirect and direct interactions between Tax and cellular molecules [6,7]. Indeed, there have been many studies showing that Tax is expressed abundantly in infected T-cells and HTLV-1-associated cell lines, and Tax acts as a main player indispensable for the malignant transformation of infected cells in the early stage of ATL development. However, ATL cells often contain genetic and epigenetic alterations of the 5'LTR of the HTLV-1 provirus, resulting in the loss of Tax expression [8]. On the other hand, the 3' end of the provirus encompassing the Tax gene is invariably maintained in leukemic cells from patients suggesting the possibility of minus strand transcription.

A novel viral protein, HTLV-1 basic zipper factor (HBZ), which is encoded by the minus strand RNA of the HTLV-1 genome, has been identified recently [9,10]. We and others identified and sequenced a novel splicing form of HBZ transcripts, named HBZ-splicing isoform (SI), which encodes a 206 amino acid protein and is generated by alternative splicing between part of the HBZ gene and a novel exon located in the 3' LTR of the HTLV-1 genome [11,12]. HBZ-SI is equivalent to the HBZ spliced variant (SPI) initiating in the 3'LTR reported by Cavanagh et al. as an alternative spliced form and to be one of the most abundant HBZ isoforms [13]. Since the spliced and non-spliced HBZ mRNAs have been reported to be detectable in almost all ATL cells tested, HBZ is expected to be closely involved in ATL cell biology corresponding to the late stages of multi-step carcinogenesis of ATL [14].

In this study, to investigate the role of HBZ in the multi-step development of ATL, the quantitative expression levels of HBZ and Tax transcripts were measured by real-time reverse-transcription PCR using HTLV-1-infected cells well characterized by HTLV-1 proviral integration status. Consequently, HBZ transcripts were observed ubiquitously in almost all cells harboring HTLV-1 provirus, and primary ATL cells were characteristic with the very high HBZ transcript levels relative to Tax.

## Materials and methods

### HTLV-1-infected cells, ATL cells, and cell lines

Blood specimens with cells carrying HTLV-1 provirus from ATL patients and healthy persons were collected in our hospital under the approval of the Research Ethics Committee of our Institute.

According to the status of cytomorphological and clinico-oncological findings, HTLV-1 proviral load and the HTLV-1 proviral integration status were determined by Southern blot analysis and classified into 31 asymptomatic carriers (AC), and 35 patients with ATL. ATL was subtyped according to the JLSG criteria [15]; acute and chronic ATL and ATL in remission. HTLV-1-related cell lines MT1, MT2, HUT102, KK1, KOB, ST1, SO4, OMT, and MT1s were examined in this study. The cell origins of MT1, KK1, KOB, ST1, SO4, and MT1s are ATL cells, whereas those of MT2, HUT102, and OMT are infected normal T-cells. MT1s is a CD4^+^T-cell line derived from MT1 during many passages [16]. All of these cell lines were documented to have 2 or more HTLV-1 proviruses monoclonally integrated into their genomic DNA. KK1, KOB, ST1, OMT, and SO4 were established in our laboratory [17,18].

HTLV-1 infection was demonstrated by a commercial anti-HTLV-1 assay kit (Fujirebio Inc. Tokyo, Japan). In this study, infected cells were defined as non-malignant T-cells randomly integrated with HTLV-1 provirus, while ATL cells were defined as malignant T-cells monoclonally integrated with the provirus.

## Methods

### DNA and RNA preparation

High molecular weight DNA was extracted from mononuclear blood cells and cell-lines using a QIAmp DNA Blood Mini kit (Qiagen GmbH, Hilden, Germany). Total RNA was extracted using ISOGEN (Nippon Gene, Toyama, Japan). After removing contaminating-genomic DNA using a Message Clean kit, two types of anti-sense cDNA and sense cDNA were synthesized. Sense cDNA was synthesized using Oligo(dT)12–18 Primer and SuperScriptTM RT (Invitrogen). The first anti-sense strand cDNAs used to amplify both HBZ and HBZ-SI mRNAs were reverse-transcribed using a minus-strand-specific primer, 5'-cccatgtctcaatactacaagaaag-3', in order to avoid contamination of cDNA from the HTLV-1 sense strand genome.

### Real-time quantitative RT-PCR for HBZ and HBZ-SI

Real-time RT-PCR was performed using a LightCycler Technology System (Roche Diagnostics) as described previously [11]. Briefly, HBZ or HBZ-SI mRNAs were amplified using anti-sense cDNA as a template and forward and reverse primers specific to the respective transcripts [11]. For the quantification of the amplicons, newly designed reporter and quencher Hybri-probes common to HBZ and HBZ-SI were used. The reporter and quencher probes were 5'-cagggctgtttcgatgcttgcctgt3'-FITC, and LC-Red-5'-tcatgcccggaggacctgctggt-3'-P, respectively. After 50 cycles, the HBZ or HBZ-SI copy number per 50 ng total RNA was estimated from the standard curves generated by serial dilution of the HBZ and HBZ-SI PCR products derived from ST1 cell line, respectively [11]. Assays were carried out in duplicate and the average value was used as absolute amounts of HBZ mRNA in samples from HTLV-1-infected individuals.

### RT-PCR quantification for Tax

The HTLV-1 Tax mRNA load was measured from a template of sense cDNA using the same LightCycler PCR System as described previously [19]. Briefly, PCR amplification was performed according to the manufacturer's instruction using the primers and probes as follows; forward primer, 5'-cccacttcccagggtttggacagag-3'; reverse primer, 5'-cgcgttatcggctcagctctcag-3', reporter probe; 5'-cttttccagaccccggactccg-3'-FITC, and quencher probe, LC-Red-5'-cccaaaacctgtacaccctctg-3'-p. After 50 cycles, the absolute amounts of HTLV-1 Tax mRNA was interpolated from the standard curves generated by the dilution method using Tax plasmids derived from a clone transfected with pGEM Easy Vector containing an amplicon of the Tax. To normalize these results for variability in RNA and cDNA integrity, we monitored abl gene in each sample as an internal control.

### SBH and HTLV-1 proviral load

Using restriction enzymes of EcoRI and PstI and a digoxigenin-labeled whole HTLV-1 probe, SBH analysis was performed as described previously [20,21]. Visible sharp band(s) from EcoRI digestion and the presence of external band(s) from PstI digestion were considered to be positive, indicating that the cells tested harbor the provirus integrated monoclonally into their genomic DNA. The detection sensitivity was at least 5%.

Next, HTLV-1 proviral load was quantified using a real-time DNA PCR LightCycler Technology System according to our previously described method [22,23]. The primers and probes used were from highly conserved sequences of the Tax gene; sense 5'-cccacttcccagggtttggacagag-3', anti-sense 5'-cgcgttatcggctcagctctcag-3', reporter probe 5'-cttttccagaccccggactccg-3'-FITC, and quencher probe LC-Red-5'-cccaaaacctgtacaccctctg-3'-P. The sample copy number was estimated by interpolation from the standard curve generated by serial dilution of a Tax-containing plasmid. The detection sensitivity was 10^-3 ^(one infected cell relative to 1000 non-infected cells). Normalization was done by using β-globin quantification as an internal control. Assuming one provirus per infected cell (one band in SBH analysis), proviral load was considered to be equivalent to the number of infected cells, namely infected-cell number per 10000 cells = (copy number of Tax)/(copy number of β-globin/2) × 10000.

### Statistical analysis

Using the Stat View software, the Mann-Whitney U test or Student's t-test were used to compare data between two groups, and Spearman's rank correlation was used to examine the two groups.

## Results

### HBZ and spliced HBZ-SI mRNA load in individuals infected with HTLV-1

The HTLV-1 proviral load represents the population of infected cells in blood mononuclear cells when one cell harbors one provirus. Accordingly, we first examined the band status of SBH analysis. Using EcoRI digested DNA samples, SBH analysis revealed one sharp band in 28 ATL samples, two bands in 2 ATL samples, and smeared-bands in 36 AC samples including 5 ATL patients in remission. The samples with two bands were adjusted when the infected cell number was estimated based on the HTLV-1 proviral load. The cell lines used here were also demonstrated to contain multiple proviruses in their genomes, e.g. 8 bands in HUT102 and 2 bands in ST1. Subsequently, the mean value of the HTLV-1 proviral load per 10^4 ^cells was 316 in ACs, 2739 in ATL patients, and 7600 in cell lines.

**Figure 1:**
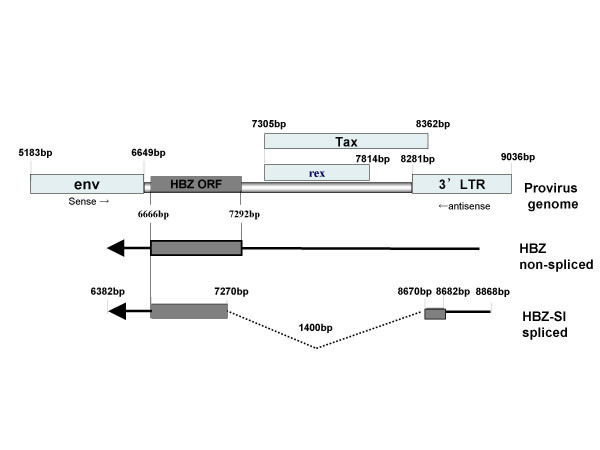
**The structure of the HBZ un-spliced (HBZ) and spliced (HBZ-SI) anti-sense transcripts (ATL-YS).** HBZ-encoding transcripts initiate in the 3'LTR and are alternatively spliced. The HBZ-SI transcript is about 2.4 kb, consisting of exon 2 corresponding to part of the HBZ ORF (7292 to 6666) and the additional exon 1 (8682 to 8670) at the 3' LTR (11). (ATL-YS; accession no. U19949).

HBZs are known to consist of non-spliced and spliced isoforms, HBZ and HBZ-SI, as shown in Figure 1. We firstly evaluated which HBZ isoform was dominant in the 54 samples from sero-positive individuals infected with HTLV-1, including 26 ACs and 28 ATL patients. The median value of un-spliced and spliced HBZ mRNA expression was 0.06 × 10^3 ^and 0.2 × 10^3 ^in ACs, 1.3 × 10^3 ^and 6.0 × 10^3 ^in ATL samples, respectively. Of all samples tested, the expression load of HBZ-SI was about 4-fold higher than that of HBZ (mean; 4.9 × 10^3 ^vs 1.2 × 10^3^; p < 0.001). Accordingly, HBZ-SI was analyzed in this study.

**Figure 2:**
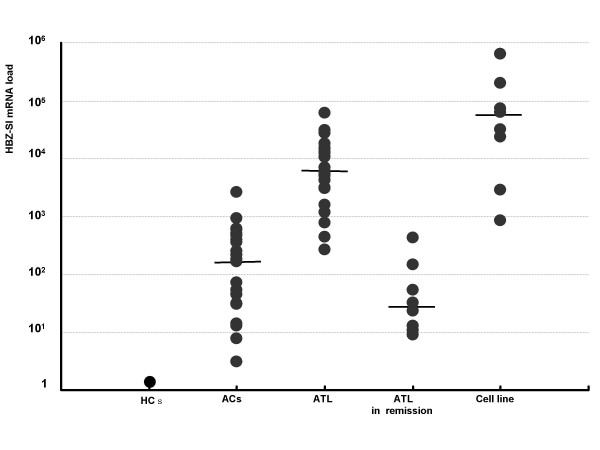
**The distribution plots of HBZ-SI mRNA load in different sample groups.** Among sero-negative controls, asymptomatic carriers (ACs), patients with ATL, ATL patients in remission, and HTLV-1-related cell lines, there is statistical difference in the median (horizontal bar) among ACs (0.2 × 10^3^) and patients withATL (6.0 × 10^3^) and cell lines (7.5 × 10^5^) (p < 0.01).

**Figure 3:**
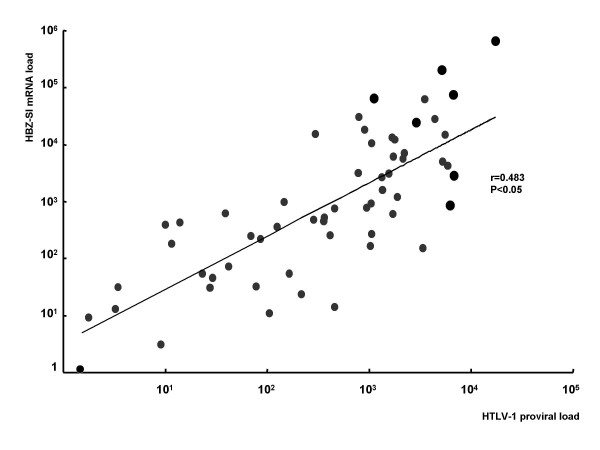
**The correlation between HBZ-SI mRNA and HTLV-1 proviral loads.** The positive correlation between the expression level of HBZ-SI mRNA (Y-axis) and the proviral load (X-axis) equivalent to the infected cell number (r = 0.483, P < 0.05), indicating that an adjusted HBZ-SI value is indispensable to evaluate the expression level in heterogeneous samples with a mixture of infected and uninfected cells.

**Figure 4:**
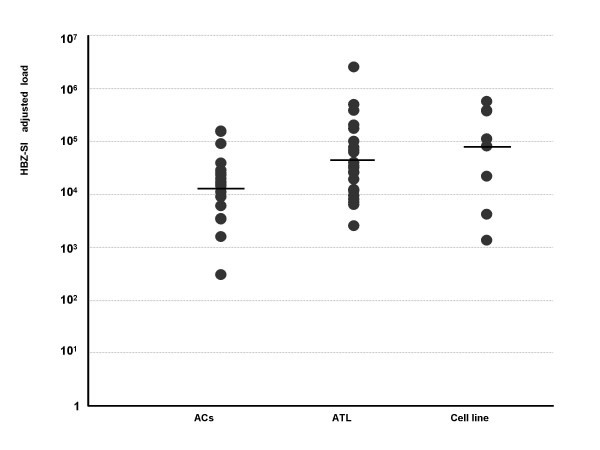
**The distribution of HBZ-SI expression level adjusted by HTLV-1 proviral load (HBZ-SI/HTLV-1 ratio) in each cell group.** The relative intensity on average among the three cell types of carriers, ATL cells from patients with ATL, and cell line cells. was about 1:6:6, but was not significantly different.

HBZ-SI mRNA load was detected in all but 5 of the 72 samples (3 ACs, one ATL, and one cell line of MT1s and ranged from 0.0 to 6.0 × 10^5^. As shown in Figure 2, the HBZ-SI mRNA load was significantly higher in ATL patients than carriers and lower in ATL patients than cell lines (p < 0.01, Mann-Whitney U-test). The relative expression load of the HBZ-SI mRNA among ACs, patients with ATL, and cell lines was 1 : 28 : 350 on average (Table 1). Furthermore, as shown in Figure 3, the HBZ-SI mRNA load was significantly correlated to the infected cell number interpolated from HTLV-1 proviral load analysis. This data reveals that, in order to understand the difference in the expression level of only provirus-positive cells, the absolute amount of HBZ-SI mRNA load should be adjusted a value per infected cell number. Accordingly, to adjust the absolute amount of HBZ-SI mRNA load in the samples consisting of a mixture of infected and non-infected cells, the proviral-adjusted HBZ-SI mRNA level (HBZ-SI/HTLV-1) was calculated as follows; (HBZ-SI mRNA load)/(HTLV-1 proviral DNA load) × 10^4^. Consequently, the HBZ-SI mRNA expression level after adjustment, as shown in Figure 4, revealed that there was a subtle difference among infected cells, ATL cells, and cell lines. The mean values of the HBZ-SI mRNA load and level before and after adjustment are summarized in Table 1, showing the changes of the relative ratio among ACs (infected cells), ATL patients (ATL cells), and cell lines, from 1 : 28 : 350 to 1 : 6 : 6.

**Table 1:** Comparison of HBZ-SI and Tax mRNA

	**ACs**	**ATL cells**	**Cell lines**	**Relative intensity^‡ ^(Acs:ATL : cell lines)**
**HBZ-SI**				
raw data*	0.04 ± 0.06	1.13 ± 1.41	14.01 ± 23.2	(1: 28: 350) ^§^
adjusted*	3.34 ± 4.71	19.82 ± 54.31	19.02 ± 22.2	(1: 6: 6)
**Tax**				
raw data*	0.009 ± 0.01	0.01 ± 0.02	590.0 ± 989.1	(1: 1: 6600) ^#^
adjusted*	0.9 ± 1.2	0.06 ± 0.01	813.0 ± 1460.0	(1: 1/15: 900) ^§^
**HBZ-SI/Tax**^†^	3.7 ± 12.0	330 ± 440^&^	0.023 ± 0.125	
				
	low HBZ/low Tax	high HBZ/low Tax	high HBZ/very high Tax	

The proviral adjusted data was calculated by dividing the raw data by HTLV-1 proviral load. Each adjusted value and HBZ-SI/Tax show the characteristic features of the mutual expression patternbetween HBZ-SI and Tax from the viewpoint of relative and absolute transcript levels. * × 104 † ratio ‡ relative expression intensity among three groups §P < 0.01 among three cell types # P < 0.01 among ATL cells and cell lines3); P < 0.01 for ACs and cell lines.

### Comparison of HBZ-SI mRNA load with Tax mRNA load in provirus-positive cells

**Figure 5:**
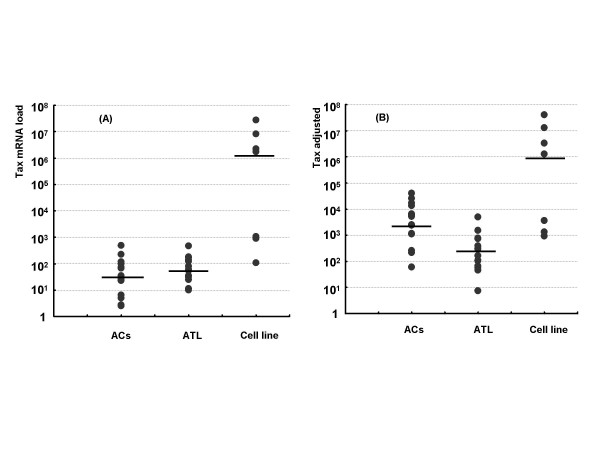
**Comparison of Tax mRNA load before (A) and after (B) adjusting by the HTLV-1 proviral load.** The data before adjustment (A) shows extremely high expression levels in only the cell lines, but that after adjustment shows apparent down-regulation in ATL cells relative to ACs and cell lines.

Tax mRNA levels were quantifiable in samples from almost all ATL patients and cell lines, and varied from 0.0 to 10^7^. However, there was no correlation between Tax mRNA load and either HBZ-SI mRNA load or proviral load. As shown in Figure 5 and Table 1, although the Tax mRNA load before adjustment was extremely high in only the cell lines, the data after adjustment (Tax/HTLV-1) clarified that ATL cells express Tax at an intensity of 15-fold less than infected cells and approximately 10^4^-fold less than cell lines.

**Figure 6:**
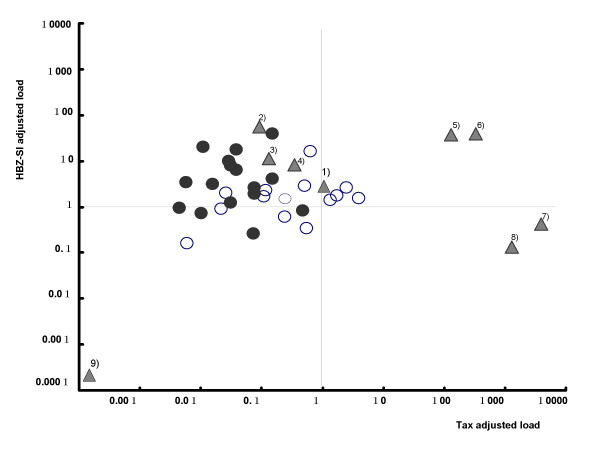
**Dot plot graph for the proviral-adjusted HBZ-SI mRNA load and the proviral-adjusted Tax mRNA load.** Each plot (Y-axis; HBZ-SI/HTLV-1 ratio, and X-axis; tax/HTLV-1 ratio) reveals the characteristic expression balance between HBZ and Tax in each cell type. ATL samples and AC samples are clustered in a low tax and high HBZ-SI area and in a central area, respectively. Of HTLV-1-related cell lines, there are two distribution types, one is ATL sample type with high HBZ-SI and low Tax, and another is a type with high Tax and either high or low HBZ-SI. Open circle; infected cells of ACs, closed circle; ATL cells, solid triangle; cell lines. ^1)^; MT-1, ^2)^; KK1, ^3)^; SO4, ^4)^; ST1, ^5)^; OMT, ^6)^; KOB, ^7)^;MT2, ^8)^; Hut102, and ^9)^;MT1s.

Then, to investigate the mutual expression status in the three provirus-positive cell types of non-malignant infected cells, ATL cells, and HTLV-1-related cell lines, the ratio of HBZ-SI/Tax was calculated. The mean ratio of HBZ-SI/Tax was 3.7 in infected cells, 330 in ATL cells, and 0.02 in the cell lines, representing an imbalanced expression between HBZ and Tax in ATL cells and cell lines compared to the base line of the 3.7 in infected cells. This feature is depicted as a twin dot plot of HBZ-SI and Tax loads in Figure 6, showing that each cell type distributes in a specific area implying the characteristic expression status of HBZ and Tax; ATL cells in an area of high HBZ-SI and low Tax, infected cells from ACs in the center area near the ATL cell area, and the majority of cell lines in an area of high Tax and either high or low HBZ. Interestingly, four cell lines (MT1, KK1, SO4, and ST1 in Fig 6 corresponding to the symbols of ^1),2),3), and 4)^) distributed in the area of high HBZ-SI and low Tax (the ATL cell area) all originated from an ATL cell clone, while three other lines (OMT, MT2, and HUT102 in Fig 6; ^5),7), and 8)^) out of 4 distributed in the area of high-Tax and either high or low-HBZ-SI were derived from infected cells.

**Figure 7:**
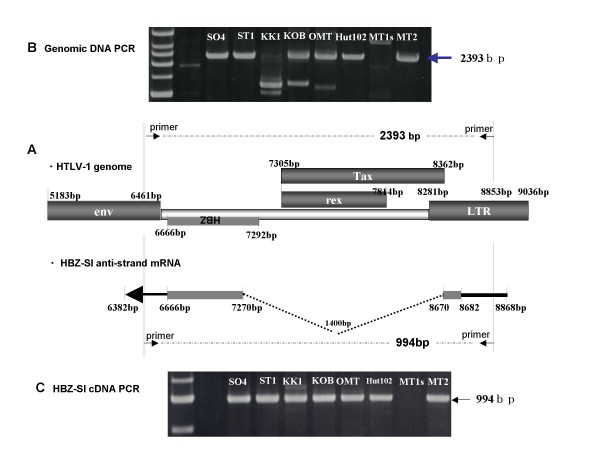
**DNA and RT-PCR analyses for the pX region including HBZ gene and for anti-sense transcript, HBZ-SI.** (A): Schematic representation of HTLV-1 genome at the position of the HBZ antisense ORF, the initiation site of the transcript, and the primer-setting positions. (B): PCR product band of the genomic region corresponding to the full-length antisense transcript. No band of the expected 2393 bp size was observed in the MT1s lane and an aberrant band was detected in the KK1 lane. (C): RT-PCR product band of anti-sense products of HBZ-SI. No band corresponding to the transcript was observed in MT1s, and only one band was visible in the other samples.

Since loss of HBZ-SI or Tax transcripts in MT1s and underestimation of proviral copy number in KK1 was observed in this study, we examined the genomic structure of the provirus by DNA PCR amplifying between nucleotides (nt) 6461 to 8853 including the region of the HBZ gene. Interestingly, as shown in panel B of Figure 7, the expected wild-type band of 2393 bp was undetectable for KK1 and MT1s, whereas MT1s, as shown in panel C, was negative for the full-length cDNA band (994 bp) derived from HBZ-SI anti-sense transcripts. These findings suggest that loss of HBZ and underestimation of the HTLV-1 proviral load could be in part explained by this genomic alteration.

## Discussion

Many studies have indicated that Tax is likely to be a central player in the induction of ATL. However, nobody has answered the paradoxical question why T-cell transformation and clonal proliferation of ATL cells is associated with a Tax-low or -negative phenotype. HBZ, a novel viral factor encoded from the minus-strand RNA of HTLV-1, is expected to play an important role in HTLV-1 biology by counteracting the action of Tax. Indeed, HBZ has been shown to interact with the cellular transcription factor CREB to inhibit HTLV-1 transcription [24]. However, there has not yet been a comprehensive study regarding the mutual expression profiles of HBZ and Tax in HTLV-1-provirus-positive cells, including infected cells, primary ATL cells, and HTLV-1-related cell lines.

This study demonstrated a ubiquitous expression of HBZ isoforms, mainly the spliced isoform of HBZ-SI, in almost all provirus-positive cells. Furthermore, in contrast to Tax, up-regulation of HBZ was characteristic of primary ATL cells, although the increase in level was subtle. These results were supported by previous studies describing that HBZ mRNA is expressed in all fresh ATL cells and HTLV-1 cell lines [11,12,25], but no quantitative observations have been reported in a large scale study. First of all, we evaluated the difference in the expression intensity between unspliced HBZ and spliced HBZ-SI (corresponding to HBZ (SP1)). Consistent with the data from a small range of samples by Cavanagh et al. [13], our results in a large range of samples clarified that HBZ-SI is the most abundant isoform, about 4-fold higher than unspliced HBZ.

Interestingly, HBZ-SI mRNA was detectable in samples from almost ACs and ATL patients. Furthermore, the HBZ-SI mRNA load was significantly correlated with the HTLV-proviral load, but not the Tax mRNA load. On the other hand, although Tax mRNA was also subtly detectable in blood samples from ACs and patients with ATL, it was not correlated with the HTLV-1 proviral load. These results indicate that the expression profile of HBZ is different from Tax, namely HBZ is near-equally expressed by all provirus-positive cells, while Tax levels are variable and can be actively up-regulated when necessary. In other words, the absolute amount of HBZ-SI mRNA load is dependent on the total of infected cells estimated by the proviral load within samples consisting of a mixture of infected and non-infected cells. Accordingly, in order to compare the expression intensity per provirus-positive cells only, it is reasonable to adjust by dividing the HBZ-SI mRNA load by the proviral copy number estimated by the HTLV-1 proviral load [26]. Actually, although the data before adjustment was generally low in ACs and patients with ATL, the adjusted data elucidated that there are no or only subtle differences in HBZ-SI expression level among infected cells, primary ATL cells, and cell lines. In particular, the relative HBZ-SI intensity of primary ATL cells and cell lines to infected cells changed from 1 : 28 : 350 before to 1 : 6 : 6 after adjustment. This up-regulation of 6-fold higher levels in primary ATL cells than infected cells is noteworthy in implication of HBZ for oncogenesis because it has been suggested that HBZ may play an important role in HTLV-1 biology by counteracting the action of Tax. Therefore, we examined the mutual expression profiles of HBZ and Tax. Our quantification analysis showed that infected cells express Tax at low levels, while primary ATL cells down-regulate Tax expression levels by 15-fold, and cell lines highly up-regulate Tax levels by 900-fold (1 : 1/15 : 900). The ratio of HBZ-SI against Tax was 4 in infected cells, 330 in primary ATL cells, and 0.02 in cell lines. Our data of the 0.02 ratio in cell lines is similar to previous data that HBZ mRNA levels are 20- to 50-fold lower (0.02) than Tax mRNA levels [12,14,27]. All of these findings indicate a characteristic imbalanced expression feature of high-HBZ and low-Tax in primary ATL cells and high-HBZ and very high-Tax in cell lines compared with a standard expression of low-HBZ and low-Tax in infected cells.

What does the difference in the mutual expression patterns, such as low-HBZ and low-Tax in infected cells, high-HBZ and no or subtle-Tax in primary ATL cells, and variable high or low HBZ and Tax in cell lines mean? Currently, only the role of Tax is stressed for oncogenic pathogenesis of HTLV-1, so the co-operative occurrence of up-regulated HBZ and down-regulated Tax may be closely associated with the oncogenic process in the early stage and with the persistent maintenance of malignancy in the late stage. Interestingly, recent reports on HBZs encoded from the minus strand of HTLV-1 seem to be providing a new insight in the current models of Tax-centered HTLV-1 pathogenesis, such as ATL oncogenesis and viral replication. In particular, the bimodal function of HBZs is of interest, in which the HBZ protein suppresses Tax transactivation of E2F1 and the HBZ mRNA promotes T-cell proliferation [12,25]. Furthermore, activation of telomerase is a critical and late event in tumor progression, HBZ also was reported to have the potential to activate telomerase through transcriptional up-regulation of hTERT by interaction with JunD and to contribute the development and maintenance of the ATL development [28]. Thus, since HBZs and Tax are thought to mutually interact with each other in the process of the multi-step oncogenesis, the imbalanced expression of HBZ and Tax in ATL cells can lead to better understand of ATL cell biology.

Another important point of this study is a mutual correlation of Tax and HBZ mRNA expression level in HTLV-1-associated cell lines. Although Tax mRNA is generally said to be low or negative in all ATL cells and extremely high in cell lines, our Tax mRNA quantification clarified that Tax mRNA was detectable in almost ATL cells at the intensity of approximately 10^4 ^fold less than that in MT2 cells, which is consistent with previous results as reported by Furukawa et al. [29]. In contrast, cell lines are known to have high levels of Tax mRNA, but our quantitative results showed that there are two types of Tax expression pattern; low Tax and high HBZ (ATL cell type) and high Tax and low (rarely high) HBZ (non-ATL cell type). This inverse correlation of Tax and HBZ expression may be explained by the HBZ function to control HTLV-1 replication as mentioned above (29). However, the existence of exceptional cases with both high Tax and HBZ expression suggests that HBZ is not everything to control Tax. Additionally, the mutual characteristic expression from cell lines appeared to correlate with its cell origin. Namely, the cell origin of cell lines having the ATL cell type of HBZ and Tax expression was a leukemic clone, while that of the non-ATL cell type was derived from HTLV-1-infected non-leukemic cells. That is, HTLV-1-related cell lines preserve the essential Tax and HBZ expression features of the original cell type.

KK1 and MT1s were found to harbor defective proviruses involving the pX/HBZ gene region, probably resulting in the loss of tax and HBZ mRNA expression in the MT1s. Despite the absence of HBZ, the cells have immortalized and survived for many generations, suggesting the possibility that HBZ may not be required in ATL cells, at least in cell lines.

In conclusion, our study provides better understanding of multi-step leukomogenesis in ATL through the characteristic expression of HBZ isoforms. Among the isoforms of HBZ, HBZ-SI is dominant over non-spliced HBZ. HBZ-SI is constantly and ubiquitously expressed in all cells harboring HTLV-1 provirus and is more highly expressed in ATL cells than in infected cells. To address ATL cell pathology induced by viral factors, it is of importance to evaluate simultaneously Tax and HBZ mRNA levels and proviral load.

## Abbreviations

HBZ: HTLV-1 basic zipper factor; SBH: Southern blot hybridization; PCR: polymerase chain reaction; AC: asymptomatic carriers; HTLV-1: human T-cell leukemia virus type-1; ATL: adult T-cell leukemia.

## Competing interests

The authors declare that they have no competing interests.

## Authors' contributions

TU designed the study, and performed the analysis. KY, KT, KM and HH recruited and monitored the subjects. YY provided the cell lines. SK made substantial contributions to the conception and design of the study, wrote and drafted the manuscript, and contributed to data interpretation.
